# Vehicle routing for a mid-day meal delivery distribution system

**DOI:** 10.1016/j.heliyon.2019.e01158

**Published:** 2019-01-29

**Authors:** Yuvraj Gajpal, Vivek Roy, B.S. Sahay

**Affiliations:** a Asper School of Business, University of Manitoba, Winnipeg, Manitoba, R3T 5V4, Canada; b Goa Institute of Management, Sanquelim, Goa, 403505, India; c Indian Institute of Management Jammu, Canal Road, Jammu, 180016, India

**Keywords:** Business, Computational mathematics

## Abstract

This paper considers the distribution system of a school feeding program (mid-day meals), wherein a set of delivery vehicles transfer cooked food from a kitchen facility to various schools within a specified delivery deadline. The food is required to be delivered before the lunch period, which is consistent across all the schools. A delay in food delivery can deprive students of their lunch, and, therefore, designing the vehicle routes for such distribution systems and maintaining a strict delivery deadline becomes critical. The resultant problem is identified as a vehicle routing problem with a common due date (VRPCDD). We provide a formulation for the VRPCDD and thereby focus on suggesting solution methods. In addition, we also demonstrate the practical application of VRPCDD by focusing on a real-life problem of a mid-day meal provider operating in the Chhattisgarh province of India.

## 1 Introduction

The recent report by “*World Food Programme*” [1, 2] recognizes school feeding programs as a big business worldwide. Intending to serve as social safety nets, these programs are helpful in promoting nutrition and education outcomes. The report specifically analyzes strengths and challenges associated with such programs for 169 countries. Thus, the report outlines the importance of gaining efficiency in these programs. We consider the distribution system for school feeding programs common in countries such as India.

In India, the school feeding program started in 2001. The program is identified as the mid-day meal scheme. It is applicable to government and government-assisted primary schools and provides cooked mid-day meals. It intends to address socio-economic objectives such as the improvement of nutritional status, enrollment, participation and retention of students in these schools [3]. World Food Programme recognized this program in India as one of the largest school feeding program in the world. The program is administered throughout the nation with the help of various mid-day meal providers (MDMP). These MDMP usually operate from a kitchen facility and use a set of delivery vans to deliver cooked food to a finite number of schools. The food is required to be delivered before the lunch time (common across all the schools). Delivery delays can deprive students of their lunch. Thus, in order to maintain a strict deadline, the total duration from the depot to the last customer of the route is restricted to the specified bound.

The problem considered in this paper is motivated by the distribution problem encountered in a mid-day meal delivery distribution system of a MDMP operating in the Chhattisgarh province of India. In the mid-day meal distribution system, all customers are required to be served before a common specified deadline time *T*. This problem is termed as vehicle routing problem with a common due date (VRPCDD). In this problem, a set of customers is served by a fleet of identical vehicles with limited capacity. The demand of the customers is known in advance. Each vehicle starts from the depot, serves a set of customers before a specified deadline and returns back to the depot. The problem determines the set of customers served by a given vehicle such that the total tour length is minimized. The total demand for any vehicle must not exceed the vehicle capacity and must ensure that customers are served before a common deadline *T*.

Furthermore, VRPCDD can be considered as a special case due to its tangential resemblance with the existing VRP structures such as Distance Constrained Vehicle Routing Problem (DVRP), Vehicle Routing Problem with Time Windows (VRPTW) and Open Vehicle Routing Problem (OVRP). As such, according to Desrochers and Laporte [4], DVRP considers the total time of any route (or total distance) to be within a pre-specified limit *L*. The term *L,* thus, applies to the total time of a vehicle route; which starts and ends at the depot. However, in VRPCDD, the total time represents the time between the depot and the last customer served, and is required to be kept within a pre-specified limit *T*. Therefore, this distinction restricts the direct application of DVRP algorithm to solve VRPCDD. For example, if the distance constraint limit *L* (of DVRP) is simply replaced by *T* (of VRPCDD), then this modification would generate infeasible solutions. To illustrate, consider a route containing only one customer *i* with travelling time *T* from the depot. This route is obviously feasible for VRPCDD. However, it is infeasible for DVRP as the total tour duration in this structure is treated as 2×T, which, in turn, violates the maximum tour time/distance constraint of DVRP. DVRP does not consider such solution components, and would therefore generate infeasible solutions for VRPCDD.

In addition, VRPCDD can be considered as a special case of VRPTW. However, the solution algorithm used to solve the VRPTW problem is inefficient, in terms of CPU time, when applied to solve VRPCDD. As such, VRPTW requires feasibility checking of individual customers to ensure the overall feasibility of a given route. In VRPCDD, checking the feasibility of the last customer is sufficient to ensure the feasibility of a given route. Thus, the VRPTW algorithm will simply spend unnecessary time in feasibility calculation of individual customers.

Additionally, VRPCDD also resembles a OVRP. However, the vehicles in OVRP either do not return to the depot or return by revisiting the assigned customers in the reverse order. This situation partially aligns with the requirements of VRPCDD. Further, OVRP algorithms cannot be employed to solve VRPCDD, but the reverse is possible.

These aspects prompted us to exclusively focus on VRPCDD. As such, in our knowledge, VRPCDD has not been studied independently. This oversight becomes important due to the practicality of VRPCDD. Mahadevan et al. [3] solve a mid-day meal distribution problem which represents the structure of VRPCDD. However, they approximate the problem as a DVRP. This observation further suggests that scholars are presently unaware of the applicability of VRPCDD. Furthermore, remaining unrecognized, the VRPCDD is being approximated with DVRP. Therefore, in this paper, we illustrate how a DVRP-based approximation can reduce the solution quality for VRPCDD.

Thus, the objectives of this paper are to introduce VRPCDD with a practical application, to provide a formulation for VRPCDD for obtaining an optimal solution, and to introduce a set of benchmark problems for future research. In doing so, we highlight the modifications needed in existing VRP algorithms for solving this problem. We further illustrate the applicability of VRPCDD with a case study which shows that a) the algorithm used for solving VRPCDD can also solve the OVRP, b) the solution of OVRP can be substantially different than the solution of closed VRP, and c) the solution quality decreases if the VRPCDD is solved as an approximation of DVRP.

The remainder of the paper is organized as follows. A literature review is presented in Section 2. Section 3 presents notations and formulation of the problem. The solution methods are presented in Section 4. In Section 5, we present numerical analysis and compare the performance of the algorithms. Conclusions follow in Section 6.

## 2 Background

The Vehicle Routing Problem (VRP) was introduced in 1959 by Dantzig and Ramser [5] to solve the Truck Dispatching Problem. In the last 55 years, a number of solution methods have been developed to solve the problem [6, 7, 8, 9, 10, 11, 12, 13, 14, 15, 16, 17, 18, 19, 20]. Laporte [21] provides an excellent overview to solve the problem by using different solution methods ranging from exact solution methods to powerful meta-heuristics. A rigorous overview of the VRP literature can be found in review works of Braekers et al. [22], Montoya-torres et al. [23] and Eksioglu et al. [24].

The notable work of Solomon and Desrosiers [25] was among the early studies to outline the importance of the VRPTW problem. The problem gained further attention due to its complexity and practical applications. These situations led researchers to propose exact algorithms [26, 27, 28, 29, 30, 31, 32]. The exact algorithms are not able to solve large problem instances, and, thus, heuristics and meta-heuristics have been extensively used for solving the VRPTW problem [33, 34]. A large number of articles in the VRPTW literature are based upon different meta-heuristics [35, 36, 37, 38, 39, 40, 41, 42, 43, 44, 45, 46, 47, 48, 49]. Important variations of the VRPTW include a vehicle routing problem in which customers require simultaneous pickup and delivery of goods during specific, individual time windows [50], a split delivery vehicle routing problem with time windows [51], and a VRP with multiple prioritized time windows [52].

An open vehicle routing problem (OVRP) is another variant of the VRP problem existing in the literature. Sariklis and Powell [53] introduced the OVRP problem with an objective of minimizing the number of routes along with the associated traveling costs. Letchford et al. [54] provide a branch-and-cut algorithm for the problem. Other solution procedures of OVRP include the use of meta-heuristics such as tabu search [55, 56], variable neighborhood search [41, 57], local search meta-heuristic [58], particle swarm optimization algorithm [59], hybrid evolution strategy [60], multi-start algorithm [61] and OVRP incorporated with cross-docking technique [62].

Numerous variations of the CVRP (Capacitated VRP), OVRP and VRPTW have been proposed: however, there are few papers that incorporate due-date features in VRP. Recently, Archetti et al. [63] and Azi et al. [64] incorporated the due-date feature in their VRP model. Archetti et al. [63] consider the multi-period vehicle routing problem with due dates (MVRPD), when customers are served between a release date and a due date. Azi et al. [64] consider the vehicle routing problem with multiple routes in which the last customer in each route is visited within a given time limit from the start of the route. In our knowledge, there is no paper in the literature that considers the special case of vehicle routing problem with a common due date.

## 3 Model

This section provides the formulation of VRPCDD, which is motivated by the MILP formulation Kulkarni and Bhave [65] provided for DVRP. The MILP formulation of Kulkarni and Bhave [65] was found to be incorrect by Achuthan and Caccetta [66] in terms of maximum tour length limit *L*. The authors found that Kulkarni and Bhave [65] did not include the depot in their constraint, and, as a result, Achuthan and Caccetta [66] added the depot in the maximum tour length constraint. Later, Naddef [67] provided another formulation based on Clarke and Wright's [68] effort to correct the formulation of Kulkarni and Bhave [65]. Therefore, in the backdrop of these corrective aspects, it is not possible to simply replace the maximum tour length limit *L* of DVRP with the common due date *T* of VRPCDD. The formulations of VRP do not capture the common due date constraint of VRPCDD, and, thereby, substantiate the novelty of the VRPCDD. Given the importance of the discussed mid-day meal distribution of VRPCDD structure, we consider the VRPCDD problem as a new variant of VRP.

The VRPCDD can be defined as follows. Given a fleet of *m* homogeneous vehicles, a set of *n* customers, let *G* = (*V*, *E*) be a complete graph where V={0,1,...,n+1} is a set of vertices and *E* = {(*i*, *j*)| *i*, *j*, ∈ *V*} is the set of arcs. The customers are represented by vertex *i* (*i* = 1,2,..,*n*), and depot is represented by the vertex 0 and *n*+1_*.*_ Vertex 0 represents the outgoing depot at which *m* identical vehicles of capacity *Q* are available. Vertex *n*+1 represents the incoming depot. With each arc (i,j)∈E i≠j, a cost *c*_*ij*_ and time *t*_*ij*_ is associated. Each customer has a non-negative demand *d*_*i*_ and a non-negative service time *s*_*i*_. In addition, each customer *i* is served before time *T* assuming that the vehicles start from the depot at time zero. The objective is to design a set of routes having a minimum total length (TTL) such that 1) each route starts and ends at the depot, 2) each customer is visited exactly once by one vehicle, 3) the total demand of the vehicle route does not exceed *Q*, and 4) customers are served (service must finish) before a deadline *T*.

Let *x*_*ijk*_ i≠j be a binary decision variable that takes value 1 if and only if arc (*i*, *j*) is used by vehicle *k* in the optimal solution and equals 0; otherwise. Let $\omega_{i}$ denote the time at which a vehicle arrives at vertex *i*. We assume that $\omega_{i}$ can take only non-negative values*.* Then, the MILP formulation of VRPCDD can be stated as follows:

(1) $$Min\;\;TTL\;\;\;=\;\;\;\sum_{i=0}^{n+1}\sum_{j=0}^{n+1}\sum_{k=1}^{m}c_{ij}x_{ijk}$$

Subject to

(2) $$\sum_{j=1}^{n+1}\sum_{k=1}^{m}x_{ijk}=1\;\;\;\;\;\;\;\;\;\;\;\;\;\;\;\;\;\;\;\;\;\;\;\;\;\;\;\;\;\;\;\;\;\;\;\;\;\;\;\;\;\;\;\;\;\;\;\forall \;i\;=1,...,n,$$

(3) $$\sum_{i=0}^{n}\sum_{k=1}^{m}x_{ijk}=1\;\;\;\;\;\;\;\;\;\;\;\;\;\;\;\;\;\;\;\;\;\;\;\;\;\;\;\;\;\;\;\;\;\;\;\;\;\;\;\;\;\;\;\;\;\;\;\;\forall \;j\;=1,...,n,$$

(4) $$\sum_{j=1}^{n+1}x_{ijk}=\sum_{j=0}^{n}x_{jik}\;\;\;\;\;\;\;\;\;\;\;\;\;\;\;\;\;\;\;\;\;\;\;\;\;\;\;\;\;\;\;\;\;\;\;\;\;\;\;\;\;\;\;\forall i=1,...,n;k=1,...,m,$$

(5) $$\sum_{j=1}^{n}x_{ojk}\leq 1\;\;\;\;\;\;\;\;\;\;\;\;\;\;\;\;\;\;\;\;\;\;\;\;\;\;\;\;\;\;\;\;\;\;\;\;\;\;\;\;\;\;\;\;\;\;\;\;\;\;\;\;\;\;\forall \;k=1,...,m,$$

(6) $$\sum_{i=1}^{n}x_{i,n+1,k}\leq 1\;\;\;\;\;\;\;\;\;\;\;\;\;\;\;\;\;\;\;\;\;\;\;\;\;\;\;\;\;\;\;\;\;\;\;\;\;\;\;\;\;\;\;\;\;\;\;\;\forall \;k=1,...,m,$$

(7) $$\sum_{i=1}^{n}d_{i}\sum_{j=1}^{n+1}x_{ijk}\leq Q\;\;\;\;\;\;\;\;\;\;\;\;\;\;\;\;\;\;\;\;\;\;\;\;\;\;\;\;\;\;\;\;\;\;\;\;\;\;\;\;\;\;\forall \;k\;=1,...,m,$$

(8) $$\omega_{i}-\omega_{j}+\left(s_{i}+t_{ij}+T\right)\sum_{k=1}^{m}x_{ijk}\leq T\;\;\;\;\;\;\;\;\;\;\forall i\;=0,...n;j\;=1,...,n\;;\;i\;\neq j,\;$$

(9) $$\;\omega_{i}\;\leq T\;\;\;\;\;\;\;\;\;\;\;\;\;\;\;\;\;\;\;\;\;\;\;\;\;\;\;\;\;\;\;\;\;\;\;\;\;\;\;\;\;\;\;\;\;\;\;\;\;\;\;\;\;\;\;\;\;\;\;\;\;\;\;\;\forall \;i\;=1...n,$$

(10) $$x_{ijk}\;\in \;\left\{0,1\right\}\;\;\;\;\;\;\;\;\;\;\;\;\;\;\;\;\;\;\;\;\;\;\;\;\;\;\;\;\;\;\;\;\;\;\;\;\;\;\;\;\;\;\;\;\;\;\;\;\;\;\;\;\;\;\;\;\forall \;i,j\;\in \;V;\;k\;=1,...,\;m;\;\;$$

(11) $$\;\omega_{i}\;\geq 0\;\;\;\;\;\;\;\;\;\;\;\;\;\;\;\;\;\;\;\;\;\;\;\;\;\;\;\;\;\;\;\;\;\;\;\;\;\;\;\;\;\;\;\;\;\;\;\;\;\;\;\;\;\;\;\;\;\;\;\;\;\;\;\;\forall \;i\;\in \;V.$$

The objective function (1) minimizes the total travel cost. The constraint (2) ensure that there is only one outgoing arc from each customer and the constraint (3) ensure that there is only one incoming arc to each customer. Constraint (4) ensure that the incoming and outgoing arc for a customer belongs to the same vehicle. Constraints (5) and (6) indicate that each vehicle must start from the outgoing depot 0 and returned to the incoming depot n+1. Constraint (7) state that a vehicle can only be loaded up to its capacity *Q*. When $\sum_{k=1}^{m}x_{ijk}=1$ (i.e., when some vehicle travels from *i* to *j*), constraint (8) impose that $\omega_{i}+s_{i}+t_{ij}\leq \omega_{j}$ for preserving route connectivity. When $\sum_{k=1}^{m}x_{ijk}=0$ (i.e., when none of the vehicle travel from *i* to *j*), constraint (8) become $\omega_{i}-\omega_{j}\leq T$ which makes the constraint redundant. Constraint (9) ensures that a customer is visited before a common due date *T*. Constraint (8) along with constraints (2), (3), (4), and (5) eliminate sub-tour constructions. Finally, constraint (10) affirm integrality constraints as binary variables and constraint (11) impose non-negativity constraint for variable$\omega_{i}$. This problem is solved using AMPL software to obtain the optimal solution.

The above formulation deviates from the formulation of DVRP with respect to constraint (8). Although there is a similarity between the common due date *T* of VRPCDD with the maximum tour length *L* of DVRP, the *T* cannot be simply substituted with *L*. The maximum tour length constraint considered by Kulkarni and Bhave [65] can be represented as follows.

(12) $$\omega_{ik}-\omega_{jk}+\left(s_{i}+t_{ij}+L\right)x_{ijk}\leq L\;\;\;\;\forall i,j\;=1,2,...n,n+1;\;\forall \;k\in \;M\;$$

Here $\omega_{ik}$ denote the time at which vehicle *k* arrives at vertex *i*. As such, the constraint (12) was considered to be incorrect by Achuthan and Caccetta [66] because node *i* does not include the depot and, thus, the first customer of the route can take the value of $\omega_{ik}$ to be zero. Therefore, these scholars suggested replacing the constraint (12) by following further two constraints to make the formulation work for DVRP.

(13) $$\omega_{ik}-\omega_{jk}+\left(s_{i}+t_{ij}+L\right)x_{ijk}\leq L\;\;\;\;\forall i,j\;=0,1,2,...n,n+1;\;\forall \;k\in \;M\;$$

(14) $$\omega_{\left(n+1\right)k}-\omega_{0k}\leq L,\;\forall \;k=1,...,m.\;$$

However, the constraints (13) and (14) will not ensure that a customer is served before a common deadline due date *T* (if *L* is simply replaced by *T*). Further, the constraints (13) and (14) are not valid inequalities for VRPCDD if *L* is simply replaced by *T*. These constraints are not satisfied for all feasible solutions of VRPCDD. Consider a VRPCDD route where a vehicle *k* starts from depot 0 and serves a customer *j* and returns back to depot n+1. Let assume that $t_{ij}=T$ and $s_{o}=0$. The values for variables $\omega_{0k}$ = 0, $\omega_{jk}$ = T and $\omega_{\left(n+1\right)k}$ = 2*T* will satisfy the VRPCDD constraint (8) because this constraint does not consider the combination between $\omega_{jk}$ and $\omega_{\left(n+1\right)k}$. However, these values will not satisfy the DVRP constraint (14). Therefore, DVRP constraints (13) and (14) become invalid inequalities for VRPCDD.

As discussed in the introduction section, the heuristic/meta-heuristic designed for DVRP cannot solve VRPCDD without modification and vice-versa. Also, the heuristic/meta-heuristic designed for OVRP cannot solve VRPCDD, but the opposite is possible. The heuristic/meta-heuristic designed for VRPTW can, however, be used to solve VRPCDD but will be inefficient. Given the above characteristics of the VRPCDD problem, we call the problem a new variant of VRP.

## 4 Methods

We propose two heuristics and one meta-heuristic to solve VRPCDD and OVRP. The first heuristic method is based on nearest neighbor, and the second heuristic is based on the well known saving heuristic of Clarke and Wright [68]. The meta-heuristic is based on tabu search.

### 4.1 Nearest neighbor (NNB) based heuristic algorithm

The nearest neighbor algorithm was one of the first heuristic algorithms used to determine a solution for the traveling salesman problem. This heuristic is easy to understand and easy to implement. This is also one of the fastest heuristics and can be implemented on a computer or simple excel sheet. In this heuristic, a salesperson starts his or her tour from a randomly chosen city and repeatedly visits the nearest city, until all cities are visited. In CVRP (Capacitated VRP), a vehicle starts its tour from the depot and successively visits the nearest customer. Thus, in each iteration, the vehicle moves from the existing customer *i* to its nearest customer *j*_._ If the visit of customer *j* violates the vehicle capacity *Q*, the vehicle returns to the depot to start the new trip. The new trip starts from the depot and visits the nearest customer of the depot_*.*_ In this way, the vehicle creates different trips, until all the customers are served. The extension of the nearest neighbor algorithm from CVRP to VRPCDD is straightforward. In VRPCDD, when a vehicle moves from an existing customer *i* to its nearest customer *j*, the common due date bound *T* is checked before moving to customer *j*_._ If the visit of customer *j* violates the common due date bound *T,* then vehicle returns back to the depot to start the new trip. In this way, the vehicle creates different trips until all the customers are served.

### 4.2 Saving (SA) based heuristic algorithm

The saving algorithm of Clarke and Wright [68] is the most widely used constructive algorithm for the variants of CVRP. The algorithm works on the principle of the maximum saving when two routes are combined and served by a single vehicle instead of two different vehicles. Consider two routes *R*_*r*_ and *R*_*q*_. Let customer *a* be the last customer of route *R*_*r*_ and customer *b* be the first customer of route *R*_*q*_. If these two routes are combined and served by a single vehicle, then the total distance traveled by the vehicle is reduced by an amount *s*_*ab*_. The reduced amount *s*_*ab*_ is called a saving which can be calculated as follows:

(15) $$s_{ab}\;=\;c_{a0}\;+\;c_{0b}\;-c_{ab}$$

Here *c*_*ab*_ is the distance between customer *a* and customer *b*. The saving algorithm of Clarke and Wright [68] for VRPCDD works as described below.

Step 1: Let *n* be the total number of customers. Assign an individual vehicle for each customer and create *n* routes as $R_{r}$ = {0, *i*, 0} for *r* = 1,…,*n.* Calculate the saving value for each combination of customers *k* and *l*, i.e., $s_{kl}\;=\;c_{k0}\;+\;c_{0l}\;-c_{kl}$ for k,l=1,...,n and k≠l. Initialize vector Ω by storing the saving values $s_{kl}$ in non-increasing order.
Step 2: Consider the first element of Ω, as $s_{kl}$ , and remove it from Ω. Determine the vehicle that serves customer *k* and customer *l.* Let customer *k* be served by vehicle *r* and customer *l* be served by vehicle *s.* Let *j* be the last customer visited by vehicle *s.* Let the total tour distance of vehicle *r* be *L*_*r*_ and the total tour distance of vehicle *s* be *L*_*s*_*.* Now check the following conditions:
  1. Customer *k* is the last visited customer in route $R_{r}$.
  2. Customer *l* is the first visited customer in route $R_{s}$.
  3. Vehicles *r* and *s* are different i.e., r≠s.
  4. The combined load of vehicles *r* and *s* is less than the vehicle capacity *Q.*
  5. If routes of vehicle *r* and *s* are combined, then the customer *j* is served before time *T* (*i.e.,* $L_{r}+L_{s}-s_{kl}-t_{j0}\;\leq \;T$).

If the above conditions are satisfied, then merge the routes of vehicles *r* and *s* and reassign vehicle *r* to serve the new route i.e., $R_{r}\;=\;R_{r}\cup R_{s}$. Label vehicle *s* as an inactive vehicle, *i*.*e*., let $R_{s}=\phi$.

Step 3. If Ω=ϕ then stop; else go to step 2.
Step 4. Return the solution $S=\left\{R_{1},R_{2},...,...,R_{v}\right\}$ where $R_{r}\neq \phi$ for *r* = 1,…,*v.*

The main difference in implementing the saving algorithm of DVRP and VRPCDD is the condition 5 of step 2. The condition 5 for VRPCDD is stated as$L_{r}+L_{s}-s_{kl}-t_{j0}\;\leq \;T$while the condition 5 for DVRP can be stated as $L_{r}+L_{s}-s_{kl}\leq \;T$*.* The numerical experiment provides the solution quality error, if the problem with VRPCDD structure is solved as a DVRP using the saving algorithm.

### 4.3 Tabu search based meta-heuristic algorithm

The tabu search heuristic is one of the most successful heuristics applied to solve VRP and its variants. Tabu search (TS) is a meta-heuristic method originally proposed by Glover [69], Glover [70] and Glover and Laguna [71]. It is an iterative procedure which explores the solution space by moving at each iteration from the current solution *s* to the best solution in its neighborhood *N*(*s*), until some stopping criterion has been satisfied. At each iteration of TS, the local transformations (or moves), that can be applied to the current solution *s*, define a set of neighboring solutions as: *N*(*s*) = {Solutions obtained by applying a single move to *s*}. The neighbor solution may be inferior to the current solution *s,* and, thus, the neighbor solution found in recent iterations is not accepted to avoid the cycle of neighborhood search. Our neighbor solution also explores the infeasible solution in terms of exceeding the vehicle capacity and due date of the last customer.

At each iteration, the best solution *s*’ in a subset V(s)⊆N(s) is selected and considered as a tabu solution for some next iterations. Tabus are stored in a short-term memory of the search (tabu list). A previously visited solution is added to the tabu list in order to prevent the repetition of solutions.

We use a unified tabu search proposed by Cordue et al. [39] to solve VRPCDD problem. An important feature of the unified tabu search is the utilization of a penalty function while allowing infeasible solutions. Consider a solution *s*, s∈S; let *c*(*s*) denote the objective function value expressed by Eq. (1). Let *q*(*s*) denote the total capacity violation of each vehicle and *w(s)* denote the total due date violation of the last customers. The total cost of the solution is then evaluated using a cost functionf(s)=c(s)+γq(s)+βw(s), where γ and β are positive weight parameters. In this equation, a penalty for constraint violation is added to the objective function *C(s)*. The penalty function allows the algorithm to search for the infeasible boundary region. The values of γ and β are dynamically adjusted to explore different regions of the search space. The overall unified tabu search algorithm as outlined by Cordue et al. [39] can be described as follows.

1. Generate a feasible solution *s* using saving algorithm described in Section 4.2.
2. Set γ=1 and β=1. Set s∗:=s and c(s∗):=c(s).
3. For *i* = 1,…, η, do the following
  • Choose a neighbor solution s'∈N(s) that minimizes f(s') and is not in the tabu list. Store solution s' in the tabu list.
  • If the solution s' is feasible andc(s')<c(s∗), set s∗:=s andc(s∗):=c(s).
  • If q(s')> 0 then set γ:=γ(1+δ); otherwise, set γ:=γ/(1+δ)
  • If w(s')> 0 then set β:=β(1+δ); otherwise, set β:=β/(1+δ)
4. Report s∗ as the final solution

The parameters are set by performing sensitivity analysis with a limited number of iterations. We set η = 10,000 iterations, penalty factor *γ* = 1, *β* = 1 and penalty multiplication factor δ = 0.5. Thus, the proposed algorithm explores the 10,000 neighbor solutions. The value of γ and β is multiplied by 1.5 if the neighbor solution is infeasible; otherwise, it is divided by 1.5.

The main difference in implementing tabu search in VRPCDD and DVRP is the generation of a neighborhood solution. The tabu search generates a neighbor solution *s’* from the existing solution *s*. When the neighbor solution is generated, DVRP requires calculating the total tour time of new route, while the VRPCDD requires the calculation of total time from the depot to the last customer of a new trip. Let us consider that neighbor solution *s’* is generated by removing customer *i* from its existing route $R_{r}$ and inserting it in the new route $R_{s}$ between customer *j* and *k*. The proposed tabu search assumes that the current solution is feasible. The route $R_{r}$ remains feasible even after removing customer *i.* Therefore, only the new route $R_{s}$ is checked for its feasibility. Let *L* and *L’* denote the total tour time of route $R_{s}$ in solution *s* and *s*’ respectively. Similarly, let *T* and *T’* denote the total tour time from the depot until the last customer of the route $R_{s}$ in solution *s* and *s’* respectively. In DVRP, the total tour length *L’* can be calculated as follows:

(16) $$L'\;=\;L-t_{jk}+t_{ji}+t_{ik}+s_{i}$$

The Eq. (16) can be used to calculate the total tour time for the new route $R_{s}$ irrespective of the place of insertion, even if the customer *v*_*i*_ is inserted just before the outgoing depot *v*_(*n*+1)._ However, this is not the case with VRPCDD. In VRPCDD, the total tour time *T’* until the last customer is served can be calculated as follows when customer *i* is inserted between customers *j* and *k* (k≠(n+1)).

(17) $$T'=T-t_{jk}+t_{ji}+t_{ik}+s_{i}$$

When customer *i* is inserted between customer *j* and outgoing depot *n*+1, the total tour time *T’* until the last customer served can be calculated as follows.

(18) $$T'=T+t_{ji}+s_{i}$$

The above expressions are valid when customer *j* is inserted in the route of another vehicle (i.e.r≠s). Similar expressions can be obtained for the case when customer *i* is inserted in another position of its existing route$R_{s}$.

## 5 Results & discussion

This section presents numerical results for the proposed algorithms and compares their performances. This section also illustrates the solution error when VRPCDD is solved as an approximation of DVRP. The algorithm described in Section 4 can solve OVRP; thus, the numerical results for an OVRP problem have also been presented. In OVRP, the vehicle starts from the depot and finishes its trip at some customer. The algorithm designed for VRPCDD can solve the OVRP by setting $c_{i\left(n+1\right)}$ = 0. The algorithm, however, cannot be used to solve VRPTW, and, therefore, comparing algorithms for VRPTW problem instances is out of the scope of this paper.

### 5.1 Case study with MDMP

We now describe the case study for the MDMP operating in a city located in the Chhattisgarh province of India. The MDMP serves the government mid-day meal initiative, which requires the serving of meals to students in government schools. The MDMP presently serves 148 schools for the morning shift (MS) and 35 schools for the day shift (DS). The cooking process takes place in a centralized kitchen facility, and the distribution is carried out through a limited number of identical vans. The distribution for the MS begins at 8:00 hrs and is required to be completed on or before 12:30 hrs. Similarly, the distribution process for the DS starts at 10:00 hrs with a deadline of 14:30 hrs. The MDMP has a fleet of 9 identical vehicles. Each of them is fitted with racks for accommodating the specially designed steel containers. When filled completely, a single container can carry food sufficient for 125 children. Every vehicle can carry a maximum of 27 containers. The container consumes the same space in the vehicle irrespective of whether a container is filled with food or empty. The number of containers represents the demand of a customer, and thus, the demand of a customer remains the same during the delivery and collection processes.

In the existing practice of MDMP, every vehicle follows a pre-determined route plan. Every route consists of 12–21 schools. Upon reaching the last school on the route, a vehicle ideally waits for a short period of time and retraces its path back in order to collect the empty vessels. However, in order to serve the DS, some of the vehicles from the MS quickly return to the depot after reaching the end of their routes. The routes for these vehicles follow the structure of VRPCDD, and the remaining vehicles follow the OVRP structure since the customer demands remain the same for the return trip.

We consider two problem instances for the case study with MS and DS with a CDD of 270 min, vehicle capacity of 27 containers and stopping time of 7 min at each school. We found the coordinate of the nodes using Google Maps and, subsequently, employed these for the calculation of inter-node distances to create X and Y coordinates for each school. The complete route for an existing solution used by MDMP is shown in Appendix. We use the existing solutions to compare our algorithms. The proposed algorithms are coded in C and implemented on AMD Opteron 2.3 GHz with 16 GB of RAM. We use an average relative percentage deviation (RPD) and absolute percentage deviation (APD) to evaluate the performance of an algorithm. The RPD and APD values are calculated as follows.

RPD={(HeuristicSolution-BestSolution)/BestSolution}×100

APD={(HeuristicSolution-OptimalSolution)/OptimalSolution}×100

We use following abbreviations for reporting results and comparing different algorithms.

TTL: Total tour length
TLULC: Total tour length up to last customer
NNB: Nearest neighborhood search based heuristic algorithm
SA: Saving based heuristic algorithm
TS: Tabu search algorithm
RPD: Relative percentage deviation
CPU: CPU time (in seconds)

Table 1 reports the result for TTL by solving the VRPCDD. The vehicles travel 496.51 kilometers in the existing solution for the MS, as used by the MDMP. NNB reduces the total distance traveled to 348.38 km, while SA reduces it to 309.25. The best solution is achieved using TS, with a 74.45 % reduction in the existing solution. The observations for DS solutions are similar to the observation of MS. Overall, TS improves the existing solution by 52.52 %.

**Table 1:** Results for TTL for the VRPCDD problem of the case study.

Instances	*N*	Existing		NNB			SA			TS		
		TTL	RPD	TTL	CPU	RPD	TTL	CPU	RPD	TTL	CPU	RPD
MS	148	496.51	74.45	348.38	<1	22.41	309.25	2.00	8.66	284.60	10.00	0.00
DS	35	47.99	30.59	46.98	<1	27.84	39.70	<1	7.96	36.77	1.00	0.00
Average		272.25	52.52	197.68	<1	25.09	174.48	2.00	8.31	160.69	5.50	0.00

Tables 2 and 3 report TLULC when problem instances are solved using the VRPCDD and OVRP structures, respectively. For solving VRPCDD, NNB provides the best solution for MS and DS with a reduction in the existing solution of 62.82 % and 11.08 % respectively. The reduction further enhances to 69.32 % (MS) and 30.49 % (DS) by TS, if the problem is solved as an OVRP.

**Table 2:** Results for TLULC for the VRPCDD problem of the case study.

Instances	*N*	Existing		NNB		SA		TS	
		TLULC	RPD	TLULC	RPD	TLULC	RPD	TLULC	RPD
MS	148	397.19	62.82	243.94	0.00	276.94	13.53	253.74	4.02
DS	35	35.39	11.08	31.86	0.00	36.61	14.91	33.66	5.65
Average		216.29	36.95	137.90	0.00	156.78	14.22	143.7	4.83

**Table 3:** Results for TLULC for the OVRP problem of the case study.

Instances	*N*	Existing		NNB		SA		TS	
		TLULC	RPD	TLULC	RPD	TLULC	RPD	TLULC	RPD
MS	148	397.19	69.32	243.57	3.83	276.94	18.06	234.58	0.00
DS	35	35.39	30.49	31.86	17.48	36.61	34.99	27.12	0.00
Average		216.29	49.91	137.71	10.655	156.78	26.525	130.85	0.00

### 5.2 Numerical experiment with benchmark problem instances

In order to evaluate the performance of the proposed algorithms more rigorously, we created more problem instances. We created two benchmark problems for VRPDD form the CVRP benchmark problems proposed by Christofides et al. [72] and Augerat et al. [73] (available at http://neo.lcc.uma.es/vrp/vrp-instances/capacitated-vrp-instances/). Furthermore, in order to obtain a contrast with the results, we also created small benchmark problem instances to get the optimal solution by evaluating the absolute performance of the proposed algorithms.

The first CVRP benchmark problem of Christofides et al. [72] consists of 14 problem instances where the number of customers varies from 50 to 199. In order to create benchmark problem for VRPCDD, we adopt only 7 problem instances where the total route duration constraint is imposed. The maximum tour length duration *L* of these problem instances are considered as a common due date (CDD) *T* to create benchmark problem for VRPCDD.

The second benchmark problem is adopted from Set A of Augerat et al. [73]. This benchmark problem consists of 27 instances with a fixed vehicle capacity of 100. The number of customers varies from 32 to 80. These problem instances do not consider service time and maximum tour length duration. We modify these problem instances to create benchmark problems for VRPCDD by adding service time and a common due date. We considered a customer's service time of $s_{i}=10,\;\forall i=1,...,n$) for all customers under all problem instances. The common due date *L* for all instances are considered to be 200.

#### 5.2.1 Absolute performance evaluation of the proposed algorithms

The small problem instances are randomly generated. The number of customers ranges from 6 to 15 with a total of 10 problem instances. The depot is located in the center of a 50 by 50 grid. The customers are randomly scattered within the grid. The demand of each customer is randomly generated between 2 to 7, and the vehicle capacity is considered to be 27.

From Table 4, it can be observed that TS closely aligns with the optimal solution (CPLEX) in terms of absolute percentage deviation (APD) and its average. The average APD of NNB and SA is 10.62 and 0.40 respectively. We also observed a larger computing time with CPLEX. These observations justify the usage of TS for solving VRPCDD.

**Table 4:** Experiment results of small instances.

Instances	n	CPLEX		NNB		SA		TS	
		TTL	CPU	TTL	APD	TTL	APD	RRL	APD
1	6	125.50	0.09	132.13	5.3	125.89	0.31	125.5	0.0
2	7	137.27	0.12	143.89	4.8	137.65	0.28	137.27	0.0
3	8	154.58	0.14	176.64	14.3	154.96	0.25	154.58	0.0
4	9	165.79	0.24	193.59	16.8	166.17	0.23	165.79	0.0
5	10	175.28	0.44	207.66	18.5	178.34	1.75	175.28	0.0
6	11	193.07	0.91	200.1	3.6	193.07	0.00	193.07	0.0
7	12	194.24	7.75	228.63	17.7	194.24	0.00	194.24	0.0
8	13	195.04	17.38	212.26	8.8	195.04	0.00	195.04	0.0
9	14	199.55	97.05	234.91	17.7	199.55	0.00	199.55	0.0
10	15	215.56	25.68	235.66	9.3	215.56	0.00	215.56	0.0
Average		170.18	14.98	189.23	10.62	170.77	0.40	170.18	0.0

#### 5.2.2 Numerical experiments with first benchmark problem instances

This section presents the result with the benchmark problem instances created by modifying the problem instances of Christofides et al. [72]. The TTL, CPU time and RPD values for the algorithms under consideration are reported in Table 5. The results reported in Table 5 show that the performance of SA is better than the performance of NNB. The average RPD of NNB and SA is 23.83 and 6.11 respectively. The comparison of CPU time indicates that the implementation of NNB is faster than SA. The performance of the tabu search meta-heuristic is much better than the heuristic solution. The solution of SA is considered as an initial solution of a tabu search (TS), and, hence, the solution of TS will be either better or equal to the solution of SA. Table 5 shows that the performance of TS is 6.11 % better than the performance of SA, although TS took more CPU time than SA. In comparison to NNB, the performance of TS is 23.83 % better. These results indicate the superiority of TS based meta-heuristic over heuristic algorithms.

**Table 5:** TTL computation by solving the VRPCDD problem with first benchmark problem instances.

Instances	*n*	NNB			SA			TS		
		TTL	CPU	RPD	TTL	CPU	RPD	TTL	CPU	RPD
C6	50	657.92	<1	20.25	598.91	<1	9.46	547.14	2	0
C7	75	1129	<1	32.30	921.91	<1	8.03	853.37	3	0
C8	100	1148.94	<1	35.67	918.59	<1	8.47	846.87	4	0
C9	150	1374.6	<1	22.74	1190.57	2	6.31	1119.93	12	0
C10	200	1669.23	<1	21.82	1432.13	7	4.52	1370.19	26	0
C13	120	1528.76	<1	4.15	1489.64	1	1.48	1467.89	7	0
C14	120	1085.15	<1	29.91	872.94	1	4.50	835.32	5	0
Average		1227.66	<1	23.83	1060.67	2.75	6.11	1005.82	8.43	0

Table 6 reports the computation results for TLULC, when the problem instances are solved using the structure of VRPCDD. Table 7 reports the TLULC when problem instances are solved using the structure of OVRP. The algorithm designed for VRPCDD can also solve the OVRP by setting $c_{i\left(n+1\right)}$ = 0. The purpose of these results is to illustrate that the algorithms designed for VRPCDD can also solve OVRP with a small modification in the code. We also want to show that when the original problem follows the structure of OVRP, then solving the problem as a closed VRP can drastically reduce the solution quality. It can be observed that by minimizing TLULC, solving the problem as an OVRP can provide better results as compared to VRPCDD. These results also reveal an interesting feature concerning the ability of heuristic algorithms (NNB and SA). The results indicate that the solution produced by heuristics (NNB and SA) are the same when problem instances are solved using VRPCDD and OVRP structures respectively. This indicates the inability of the two heuristics to distinguish between the structure of VRPCDD and OVRP. However, TS is effective in overcoming this limitation. The TS provides a better solution when the problem is solved as an OVRP.

**Table 6:** TLULC computation by solving the VRPCDD problem for first benchmark problem instances.

Instances	*n*	NNB		SA		TS	
		TLULC	RPD	TLULC	RPD	TLULC	RPD
C6	50	469.82	0.00	502.27	6.91	477.01	1.53
C7	75	751.53	13.54	715.71	8.13	661.89	0.00
C8	100	817.22	11.54	770.61	5.18	732.65	0.00
C9	150	936.92	2.81	946.44	3.85	911.31	0.00
C10	200	1080.25	0.00	1096.43	1.50	1118.02	3.50
C13	120	953.17	0.00	1050.54	10.22	1005.68	5.51
C14	120	714.97	17.47	608.63	0.00	622.26	2.24
Average		817.70	6.48	812.95	5.11	789.83	1.83

**Table 7:** TLULC computation by solving the OVRP problem for first benchmark problem instances.

Instances	*n*	NNB		SA		TS	
		TLULC	RPD	TLULC	RPD	TLULC	RPD
C6	50	469.82	12.93	502.27	20.73	416.04	0.00
C7	75	751.53	32.40	715.71	26.09	567.64	0.00
C8	100	817.22	22.90	770.61	15.89	664.93	0.00
C9	150	936.92	19.62	946.44	20.83	783.26	0.00
C10	200	1080.25	17.34	1096.43	19.10	920.58	0.00
C13	120	953.17	2.93	1050.54	13.45	926.01	0.00
C14	120	714.97	25.03	608.63	6.43	571.86	0.00
Average		817.70	19.02	812.95	17.50	692.90	0.00

#### 5.2.3 Numerical experiments with second benchmark problem instances

This section presents the results of a numerical experiment with second, benchmark problem instances created by modifying the benchmark problem instances of Augerat et al. [73]. Table 8 reports TTL by solving the problem instances as a VRPCDD structure. The results are similar to the results of the first benchmark problem instances. The performance of NNB is observed to be worse, while the performance of TS is the best. The performance of TS is 26.28 % better than the performance of NNB and 7 % better than the performance of SA. Other observations are similar to the observations with first benchmark problem instances. We notice similar observations as first benchmark problem instances when the problem instances are solved using the structure of VRPCDD and OVRP. Results, however, are not being reported to keep the paper concise.

**Table 8:** TTL computation by solving the VRPCDD problem with second benchmark problem instances.

Instances	*n*	NNB			SA			TS		
		TTL	CPU	RPD	TTL	CPU	RPD	TTL	CPU	RPD
A-n32-k5	31	1107.24	<1	28.96	972.61	<1	13.28	858.59	<1	0.00
A-n33-k5	32	733.15	<1	7.73	722.55	<1	6.17	680.54	<1	0.00
A-n33-k6	32	1040.78	<1	40.14	795.67	<1	7.13	742.69	<1	0.00
A-n34-k5	33	955.75	<1	19.67	817.55	<1	2.37	798.64	<1	0.00
A-n36-k5	35	1036.79	<1	15.50	984.23	<1	9.64	897.67	<1	0.00
A-n37-k5	36	951.97	<1	31.16	775.60	<1	6.86	725.80	<1	0.00
A-n37-k6	36	1236.94	<1	22.64	1126.84	<1	11.72	1008.61	<1	0.00
A-n38-k5	37	1070.95	<1	38.42	806.24	<1	4.21	773.68	<1	0.00
A-n39-k5	38	1081.59	<1	18.61	975.54	<1	6.98	911.91	<1	0.00
A-n39-k6	38	1160.59	<1	32.25	973.73	<1	10.96	877.55	<1	0.00
A-n44-k7	43	1333.98	<1	31.05	1081.11	<1	6.20	1017.95	1.00	0.00
A-n45-k6	44	1335.35	<1	33.29	1069.85	<1	6.79	1001.83	1.00	0.00
A-n45-k7	44	1457.24	<1	23.83	1259.60	<1	7.04	1176.76	1.00	0.00
A-n46-k7	45	1383.20	<1	43.73	984.31	<1	2.28	962.38	1.00	0.00
A-n48-k7	47	1434.05	<1	22.14	1306.84	<1	11.31	1174.10	1.00	0.00
A-n53-k7	52	1314.31	<1	19.12	1149.69	<1	4.20	1103.37	1.00	0.00
A-n54-k7	53	1606.78	<1	33.79	1317.19	<1	9.68	1200.96	2.00	0.00
A-n55-k9	54	1379.59	<1	28.40	1110.57	<1	3.36	1074.46	1.00	0.00
A-n60-k9	59	1665.55	<1	21.61	1497.34	<1	9.33	1369.56	2.00	0.00
A-n61-k9	60	1308.09	<1	25.42	1103.90	<1	5.84	1042.96	2.00	0.00
A-n62-k8	61	1827.01	<1	28.49	1505.59	<1	5.88	1421.95	6.00	0.00
A-n63-k9	62	2147.49	<1	20.50	1843.85	<1	3.47	1782.10	2.00	0.00
A-n63-k10	62	1867.55	<1	39.11	1414.39	<1	5.36	1342.48	2.00	0.00
A-n64-k9	63	1829.93	<1	17.23	1773.15	<1	13.59	1561.00	2.00	0.00
A-n65-k9	64	1453.15	<1	22.66	1243.39	<1	4.96	1184.66	2.00	0.00
A-n69-k9	68	1476.26	<1	23.69	1268.35	<1	6.27	1193.53	2.00	0.00
A-n80-k10	79	2551.13	<1	20.36	2208.86	<1	4.21	2119.63	3.00	0.00
Average		1398.02	<1	26.28	1188.46	<1	7.00	1111.31	1.88	0.00

#### 5.2.4 Effect of approximation of VRPCDD with DVRP

This section presents the results of a numerical experiment with first and second benchmark problem instances when DVRP is used to approximate VRPCDD. The common due date value of *T* in VRPCDD is considered as the maximum tour time value *L* for DVRP. It is obvious that the objective function value (i.e., the total distances traveled by all the vehicles) will deteriorate when *L* is replaced by *T*. The purpose of this analysis is to observe the magnitude of such deterioration. We modify the algorithm designed for VRPCDD to solve a DVRP problem. The NNB and SA can be easily modified to solve the DVRP; however, it is not easy to modify the tabu search designed for VRPCDD. We, therefore, report the solutions of NNB and SA only. The problem is first solved as VRPCDD and total distances are reported under the heading “*TTL for VRPCDD*”. The solution reported under heading “*TTL for DVRP*” reports the solution when the value of *T* is considered as maximum tour time value *L* for DVRP. The results for first benchmark problem instances are reported in Table 9 and show that this approximation reduces the quality of solution (as indicated by percentage error of 10.82 % for NNB and 5.5 % for SA). Table 10 reports the same for second benchmark problem instances and reports the percentage error as 22.28 % for NNB and 22.18 % for SA. Some of the problem instances results are not reported in Table 10 because problem instances become infeasible for DVRP due to tight common due date. The results in Tables 9 and 10 also indicate that the approximation error becomes substantially large for tight common due date instances. Given the application of the structure in the mid-day meal distribution system, Mahadevan et al. (2013) solve VRPCDD using DVRP. The presence of error in such approximations, therefore, will yield solutions which can be substantially improved. In the specific application to the mid-day meal distribution system, the maximum tour time is usually restricted to 2–4 hours. Given the shortness of the travel span, an approximation of the system using DVRP can be an expensive prospect.

**Table 9:** Effect on approximation of VRPCDD with DVRP for first benchmark problem instances.

Instances	*n*	NNB			SA		
		TTL for DVRP	TTL for VRPCDD	Percentage Error	TTL for DVRP	TTL for VRPCDD	Percentage Error
C6	50	784.50	657.92	19.24	622.63	598.91	3.96
C7	75	1192.23	1129.00	5.60	972.39	921.91	5.48
C8	100	1260.58	1148.94	9.72	963.67	918.59	4.91
C9	150	1471.00	1374.60	7.01	1300.82	1190.57	9.26
C10	200	1753.78	1669.23	5.07	1543.74	1432.13	7.79
C13	120	1904.05	1528.76	24.55	1587.88	1489.64	6.59
C14	120	1134.78	1085.15	4.57	877.06	872.94	0.47
Average		1357.27	1227.66	10.82	1124.03	1060.67	5.50

**Table 10:** Effect on approximation of VRPCDD with DVRP for second benchmark problem instances.

Instances	*n*	NNB			SA		
		TTL for DVRP	TTL for VRPCDD	Percentage Error	TTL for DVRP	TTL for VRPCDD	Percentage Error
A-n33-k5	32	1066.30	733.15	45.44	779.69	722.55	7.91
A-n33-k6	32	1249.44	1040.78	20.05	821.63	795.67	3.26
A-n34-k5	33	1139.58	955.75	19.23	1036.35	817.55	26.76
A-n37-k5	36	1150.36	951.97	20.84	908.50	775.60	17.14
A-n38-k5	37	1099.83	1070.95	2.70	898.19	806.24	11.40
A-n39-k6	38	1501.30	1160.59	29.36	1258.91	973.73	29.29
A-n45-k6	44	2013.31	1335.35	50.77	1664.21	1069.85	55.56
A-n46-k7	45	1687.25	1383.20	21.98	1362.78	984.31	38.45
A-n55-k9	54	1340.93	1379.59	-2.80	1256.81	1110.57	13.17
A-n61-k9	60	1496.84	1308.09	14.43	1182.06	1103.90	7.08
A-n63-k10	62	2368.39	1867.55	26.82	2005.93	1414.39	41.82
A-n65-k9	64	1842.47	1453.15	26.79	1563.24	1243.39	25.72
A-n69-k9	68	1682.83	1476.26	13.99	1405.71	1268.35	10.83
Average		1510.68	1239.72	22.28	1241.85	1006.62	22.18

## 6 Conclusion

We have studied a vehicle routing problem with a common due date (VRPCDD), in which all customers are served before a commonly specified deadline. The problem is shaped by the mid-day meal distribution system in which food is delivered before the lunch time at the schools. We have developed two heuristic algorithms to solve the problem. The first heuristic algorithm is based on nearest neighborhood search, and the second heuristic is based on a well known saving algorithm. We have also developed a tabu search based meta-heuristic to achieve better solutions. The comparisons of the algorithms are performed on new benchmark problem instances. The new problem instances are created by modifying the benchmark problem instances of CVRP. The numerical experiments indicate that NNB is quickest in producing results, but the solution quality is inferior. The SA provides a better solution in comparison to NNB but consumes more CPU time. The best result is obtained by TS, but the CPU time consumption is more than the CPU time consumption of heuristic algorithms. The numerical results also reveal that solving VRPCDD with an approximation of DVRP can substantially deteriorate the solution up to 22 %. The application of VRPCDD is demonstrated by solving the problem instances of a mid-day meal provider (MDMP) operating in the Chhattisgarh province of India. The proposed tabu search algorithm improves the existing solution of MDMP by 52.52 % for VRPCDD and 49.91 % for OVRP.

## Declarations

### Author contribution statement

Yuvraj Gajpal: Conceived and designed the experiments; Performed the experiments; Analyzed and interpreted the data; Contributed reagents, materials, analysis tools or data; Wrote the paper.

Vivek Roy: Analyzed and interpreted the data; Contributed reagents, materials, analysis tools or data; Wrote the paper.

B.S. Sahay: Contributed reagents, materials, analysis tools or data; Wrote the paper.

### Funding statement

This work was supported by NSERC discovery (Grant 318689).

### Competing interest statement

The authors declare no conflict of interest.

### Additional information

No additional information is available for this paper.
