# Supplementary material for: Vehicle routing for a mid-day meal delivery distribution system
Source: Heliyon. 2019 Jan 29;5(1):e01158. doi: 10.1016/j.heliyon.2019.e01158 (PMC6356091; doi:10.1016/j.heliyon.2019.e01158)
Supplement: Appendix [file mmc1.docx]

**Appendix**

Existing Solution as followed by the mid-day meal provider (MDMP) described in Section 5.1. In the solution, “0” indicates the depot and positive number indicates the customers.

**Existing Solution for MS instances of MDMP with total distance of 496.51:**

1: 0-1-2-3-4-5-6-7-8-9-10-11-12-13-14-15-16-17-0

2: 0-18-19-20-21-22-23-24-25-26-27-28-29-30-31-32-33-0

3: 0-34-35-36-37-38-39-40-41-42-43-44-45-46-47-48-49-50-51-0

4: 0-52-53-54-55-56-57-58-59-60-61-62-63-0

5: 0-64-65-66-67-68-69-70-71-72-73-74-75-76-0

6: 0-77-78-79-80-81-82-83-84-85-86-87-88-89-90-91-92-93-94-95-0

7: 0-96-97-98-99-100-101-102-103-104-105-106-107-108-109-110-111-112-113-114-115-116-0

8: 0-117-118-119-120-121-122-123-124-125-126-127-128-129-130-0

9: 0-131-132-133-134-135-136-137-138-139-140-141-142-143-144-145-146-147-148-0

**Existing Solution for DS instances of MDMP with total distance 47.99:**

10: 0-149-150-151-152-153-154-155-156-157-158-159-160-161-162-163-164-165-166-0

11: 0-167-168-169-170-171-172-173-174-175-176-177-178-179-180-181-182-183-0

**Tabu Search Solution for MS instances of MDMP with total distance 284.60:**

1: 0-55-56-96-63-62-59-104-105-93-91-92-61-60-57-53-66-65- 0

2: 0-3-40-41-42-11-80-44-43-48-45-0

3: 0-8-103-5-6-7-9-131-132-133-116-10-115-14-15-13-107-106-100-102-101-77- 0

4: 0-2-31-30-27-29-28-23-21-22-24-20-25-18-19-51-47-50-46-49-34-0

5: 0-58-81-83-84-85-108-109-86-82-88-87-113-112-110-111-95-94-89-90-16-67- 47-6-0

6: 0-52-69-68-54-64-70-71-72-73-74-75-79-99-98-97-78-39-38-37-35-36-0

7: 0-26-32-129-130-140-137-138-141-139-128-127-126-124-125-123-121-0

8: 0-33-147-148-143-144-142-146-145-120-122-119-118-117-17-136-135-12-134-114-1-0

**Tabu Search for DS instances of MDMP with total distance 36.77:**

1: 0-20-21-22-23-4-1-5-6-7-8-27-35-34-33-32-31-30-29-28-26-24-0

2: 0-13-12-18-17-15-14-16-10-11-23-9-25-19-0
